# Hotel Dieu

**Published:** 1831-02

**Authors:** 


### HOTEL DIEU.

### *Large Tumor growing from the Side of the Thyroid Gland; Ligature of the Pedicle; Death in eighteen Hours. Cases in which fatal Results have been produced by the Entrance of Air into the Veins.**

A BOY, twelve years of age, of good constitution, had, from his earliest infancy, the nucleus of a tumor on the left side of the neck, which had gradually increased till it equalled the head in size; in which state he was admitted into the Hôtel Dieu in October. There was no change in the appearance of the skin, the tumor occupied the entire left side of the neck, and appeared to have a pedicle; behind it could be felt the carotid artery; above and beneath, the pulsation of the superior and inferior thyroids was perceptible; the tumor was hard, elastic, and covered with large veins, one of which

* Medical Gazette.

was very voluminous, and ran obliquely from above downwards and from without inwards, so that a longitudinal incision could not be made without dividing it. M. Dupuytren hesitated for some time before he made up his mind to any operation: he remembered the unfortunate result which happened in the case of a young girl in whom he extirpated a large tumor from the neck some time ago.—[The dissection was almost completed, when, on lifting up the tumor to finish it, a rushing noise was heard, the patient uttered a cry, fell senseless, and, after some convulsions, expired in the theatre. Post-mortem examination demonstrated the entrance of air into the veins and right ventricle of the heart; and it was to the admission of this through a divided vein that M. Dupuytren attributed the death of his patient.] The fear of this kind of accident, of which Graefe has since seen an instance, and which has also been observed by M. Clemot de Rochefort, influenced him in postponing an operation.

The circumstances, as mentioned by M. Clemot, are these. In the dissection of a tumor from the axilla, all at once a noise was heard like that of blowing, or respiration; the assistants imagined that M. Clemot had penetrated the chest, and the patient (a female), after a lively exclamation of complaint, fell into a state of syncope. After a time, she recovered from the fainting, and M. Clemot tied the vein by which the air had entered. On another occasion, in tying the subclavian after the division of a small vein, a feeble but distinct sound of "aspiration" was heard: M. Clemot put his finger on the vein, and the sound ceased; he removed his finger, and it was renewed. A ligature was applied to the vessel, and no inconvenience resulted. A third time, in the extirpation of a tumor from the breast, which weighed twelve pounds, a similar accident occurred to that which took place in the hands of Dupuytren and Graefe, the patient dying in the course of a few hours.

On examination after death in this instance also, the veins leading from the wound to the heart, the right auricle and ventricle, were distended with air. In the case, however, which more particularly forms the subject of the present article, the patient was in good health, and the tumor of a nature inevitably to produce fatal consequences if allowed to continue its growth; and M. Dupuytren resolved to operate, taking especial care to avoid the danger above described by making assistants place their fingers in the openings of the vessels at the moment when the raising of the tumor in dissecting it out should have a tendency to produce that kind of suction by which air might be drawn into the veins.

Nov. 22. M. Clemot, pressing both hands on each side of the base of the tumor, so as to make it project, a longitudinal incision was made through the skin and integuments, along the whole of the anterior part of the tumor; after which, by a sort of turning out, and dissection of the morbid growth, the pedicle was exposed. This proved to be of such magnitude that it was not deemed prudent to carry the incisions farther. At the commencement some veins had bled, and M. Breschet had applied his fingers upon the orifices; two or three small arteries had also bled, and been tied. A bistoury was now plunged into the tumor, in the hope of diminishing its size by giving issue to the fluid which it was supposed to contain: nothing, however, escaped but a little blood. Hemorrhage now occurring from numerous points, and the pedicle being too extensive to be divided, a ligature was applied around it, followed by a second and a third. The compression thus effected soon stopped the jets of arterial blood, but not the venous oozing: however, after a little time, this also nearly ceased, and upon the whole the loss was not very great. The patient fainted, but was easily recovered from this by sprinkling cold water on the surface, and he was replaced in bed; it being intended to substitute a metallic ligature for the thread, with the view of commanding the bleeding more effectually, and also of cutting through the pedicle. Two pupils were placed to watch him.

During the day obstinate vomiting took place, which was not arrested by Seltzer water, but which ceased towards evening. The patient lost but little blood during the day, and he appeared to have escaped from the immediate dangers of the operation, when, about three o'clock in the morning, he was seized with convulsions, and expired. It ought to be stated, that, several times during the operation, the patient had complained of choking when any pressure was made on the tumor on the side next the larynx; and this inconvenience ceased when such pressure was removed. What produced the vomiting? It might have been apprehended that the pneumogastric nerve was cut, or included in the ligature, but the post-mortem examination showed that such was not the case.

The tumor proved to be composed of fibro-cellular tissue, partly "degenerated," and of a brownish colour. The trachea was flattened, from the pressure which had been made upon it. The carotid-jugular veins, and thyroideal arteries, were entire. The tumor had its origin at the left side of the thyroid gland.

M. Dupuytren remarked, that in a parallel case he should probably abstain from operating; for he admitted that the ligature had produced the fatal symptoms and death, while he believed that the incision of the pedicle would have immediately proved fatal by hemorrhage.—*Lancette Française.*

It appears not a little extraordinary that the narrator of the above case should ask what produced the vomiting? It is quite obvious that it was the sympathetic result of the ligature applied round the pedicle of the tumor, and was part of the disturbance which was caused by this source of irritation; a source to which the death of the patient is to be attributed. Would it not have been better to have cut out the tumor at all hazards, and taken the chance of being able to arrest the hemorrhage?

